# Supplementary material for: Optimization of Enterovirus-like Particle Production and Purification Using Design of Experiments
Source: Pathogens. 2025 Jan 27;14(2):118. doi: 10.3390/pathogens14020118 (PMC11858008; doi:10.3390/pathogens14020118)

Figure 2A

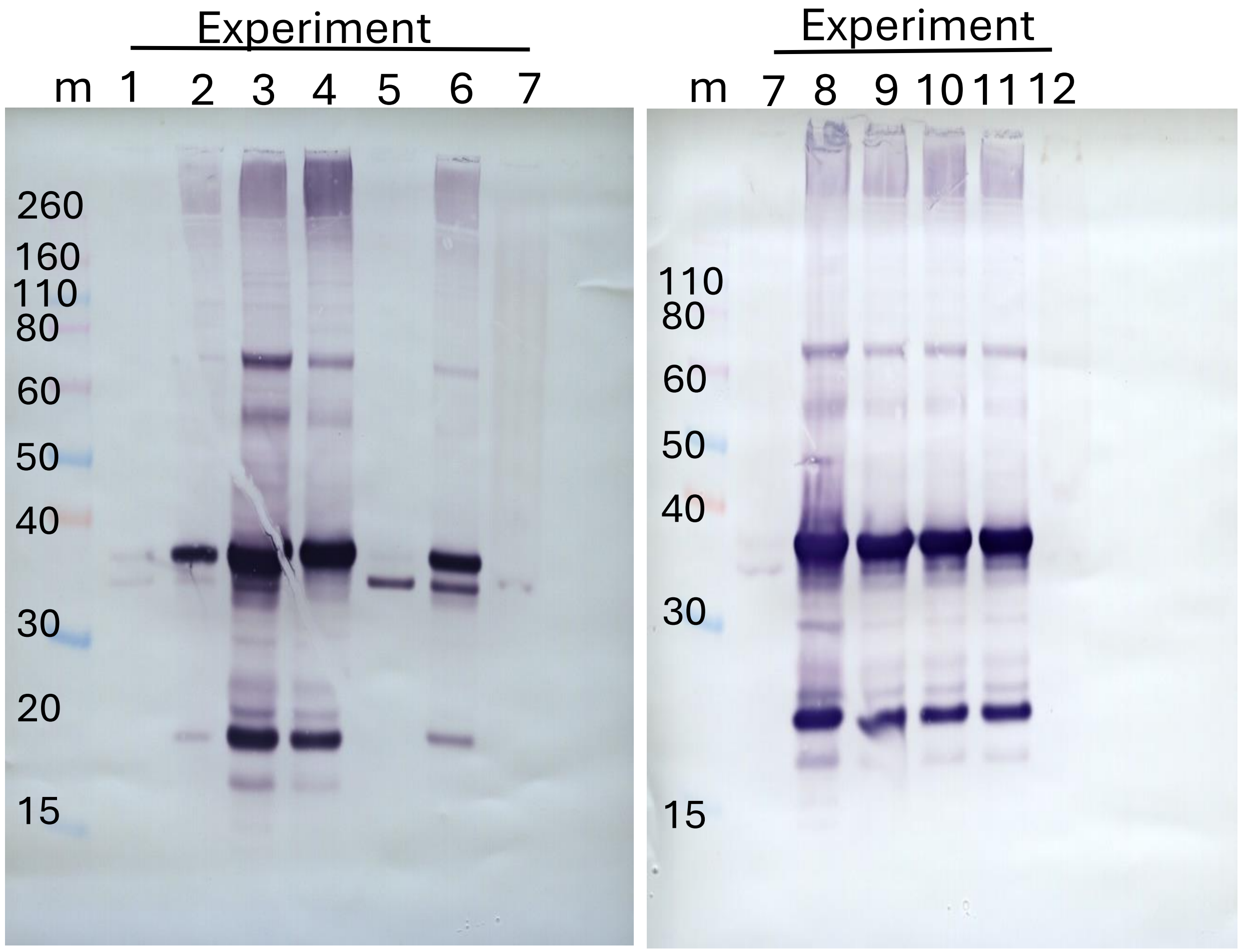
Experiment
m 1 2 3 4 5 6 7
260
160
110
80
60
50
40
30
20
15
Experiment
m 7 8 9 10 11 12
110
80
60
50
40
30
15

Figure 2B

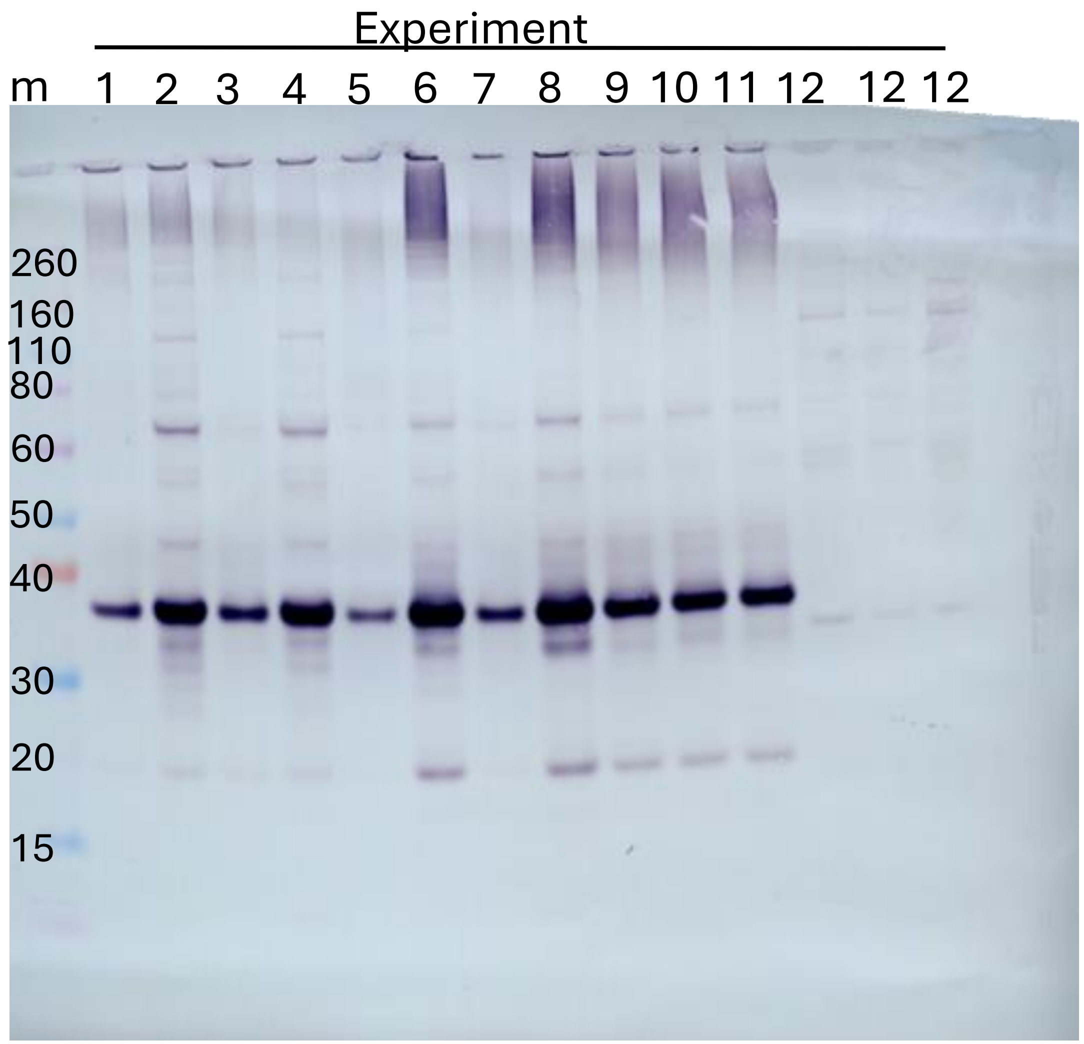

Experiment
m 1 2 3 4 5 6 7 8 9 10 11 12 12 12
260
160
110
80
60
50
40
30
20
15

Figure 6 - SDS

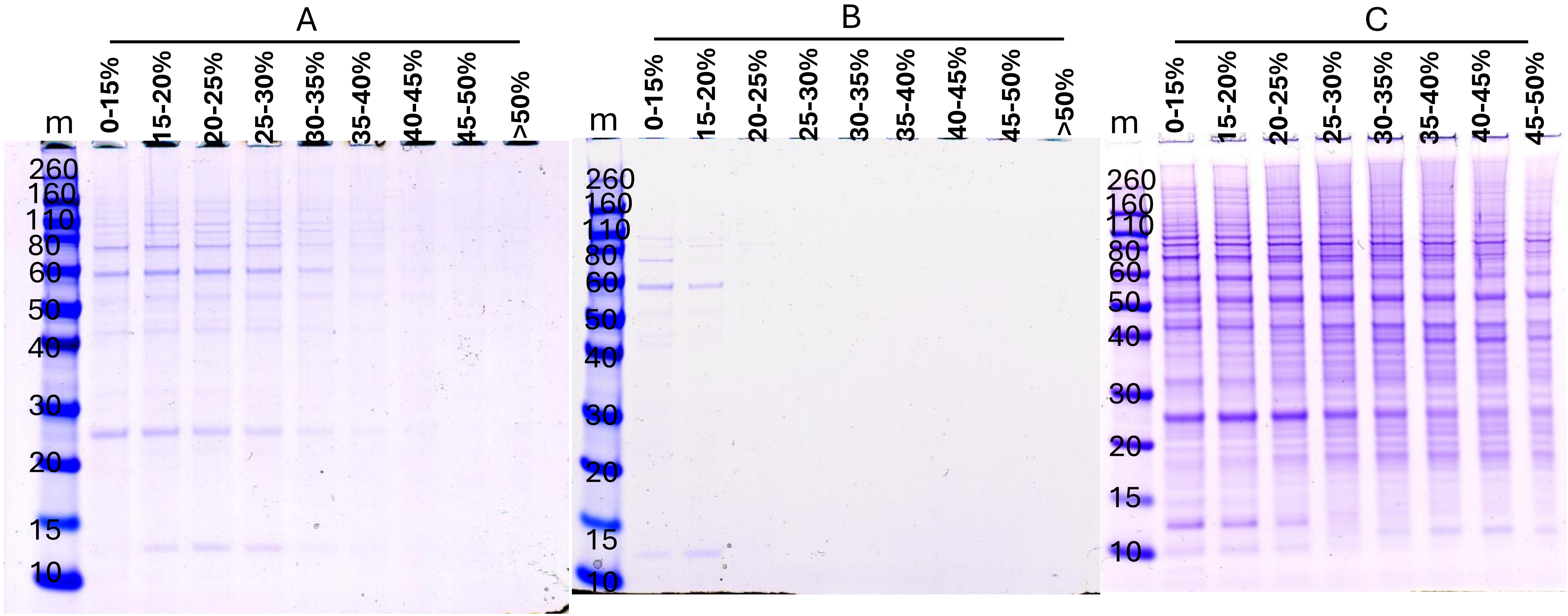
A
B
C
m
0-15%
15-20%
20-25%
25-30%
30-35%
35-40%
40-45%
45-50%
>50%
260
160
110
80
60
50
40
30
20
15
10

Figure 6 - SDS

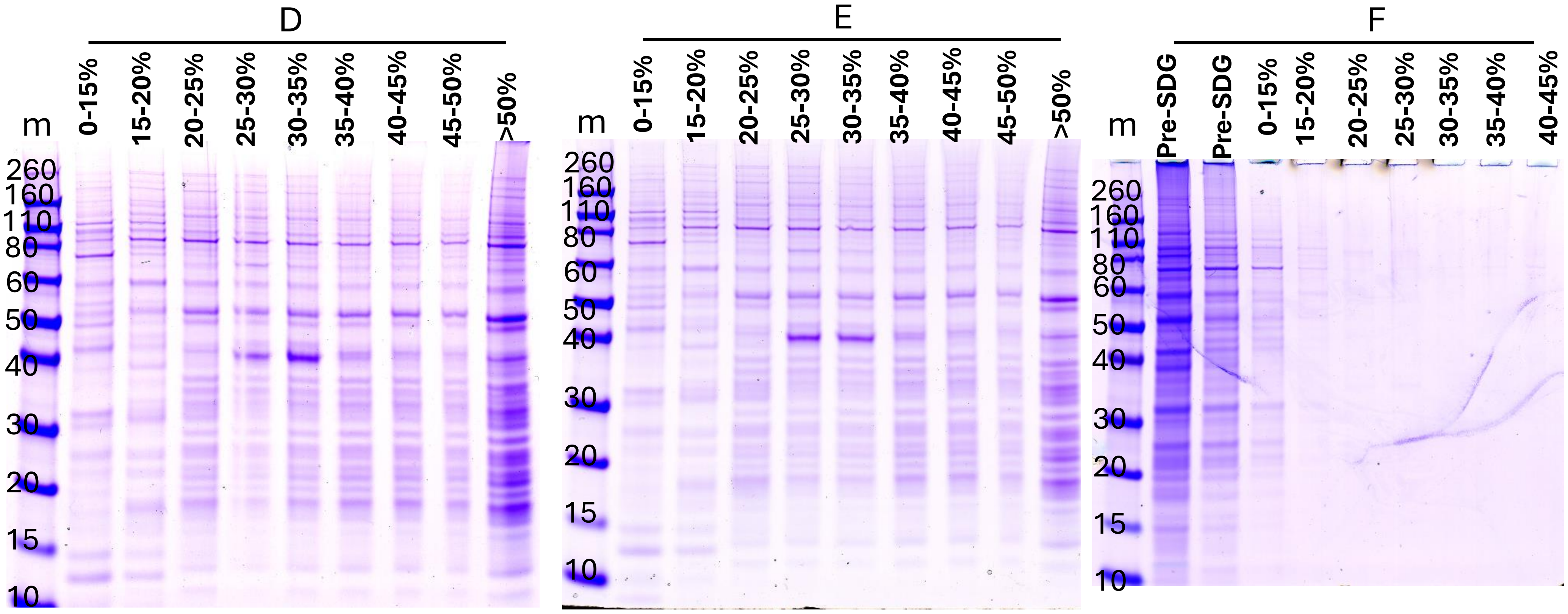

D
m
0-15%
15-20%
20-25%
25-30%
30-35%
35-40%
40-45%
45-50%
>50%
260
160
110
80
60
50
40
30
20
15
10
E
m
0-15%
15-20%
20-25%
25-30%
30-35%
35-40%
40-45%
45-50%
>50%
260
160
110
80
60
50
40
30
20
15
10
F
m
Pre-SDG
Pre-SDG
0-15%
15-20%
20-25%
25-30%
30-35%
35-40%
40-45%
260
160
110
80
60
50
40
30
20
15
10

Figure 6 – western blot

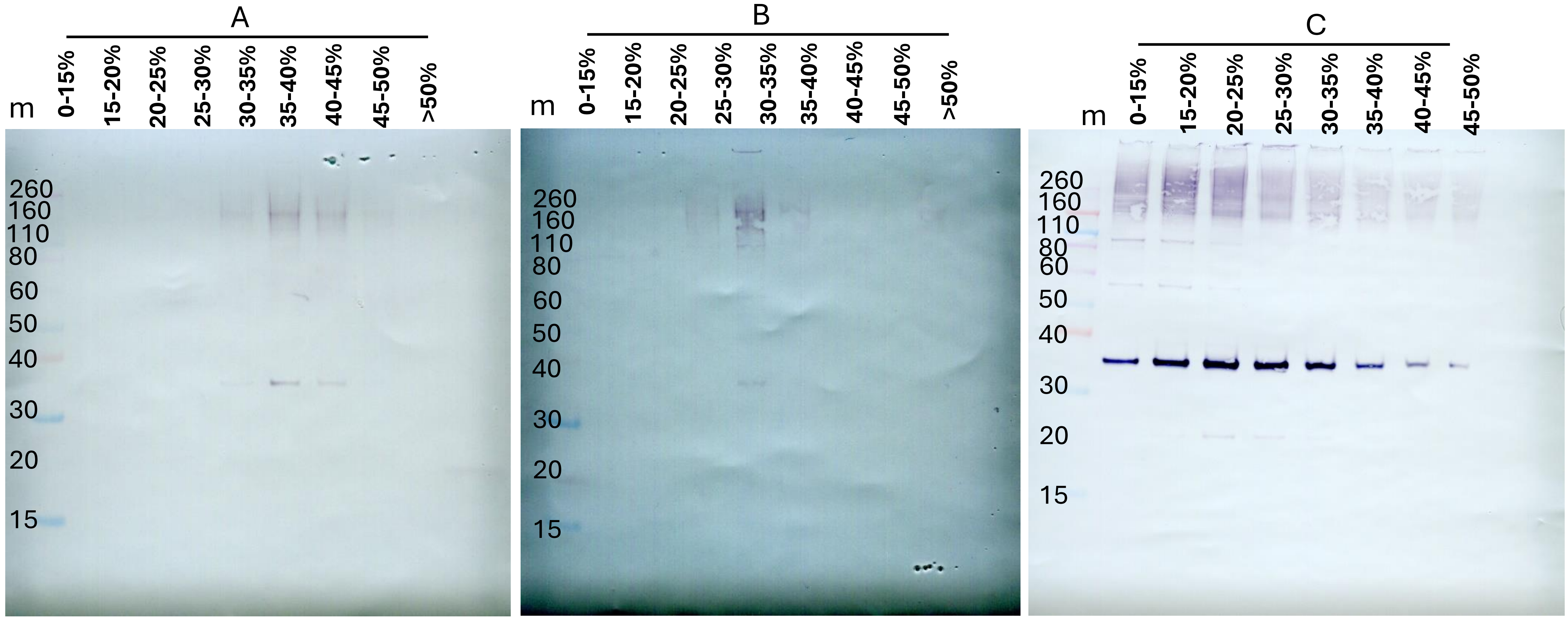

Figure 6 – western blot

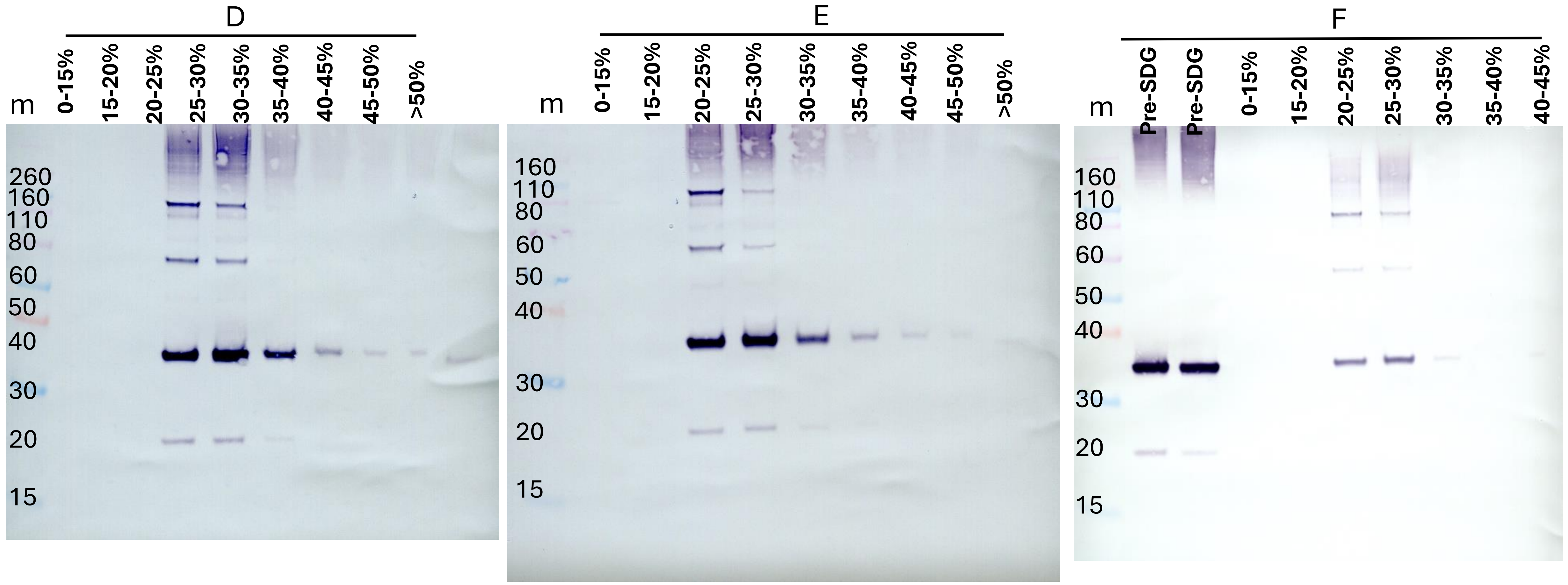

Figure 8B - SDS

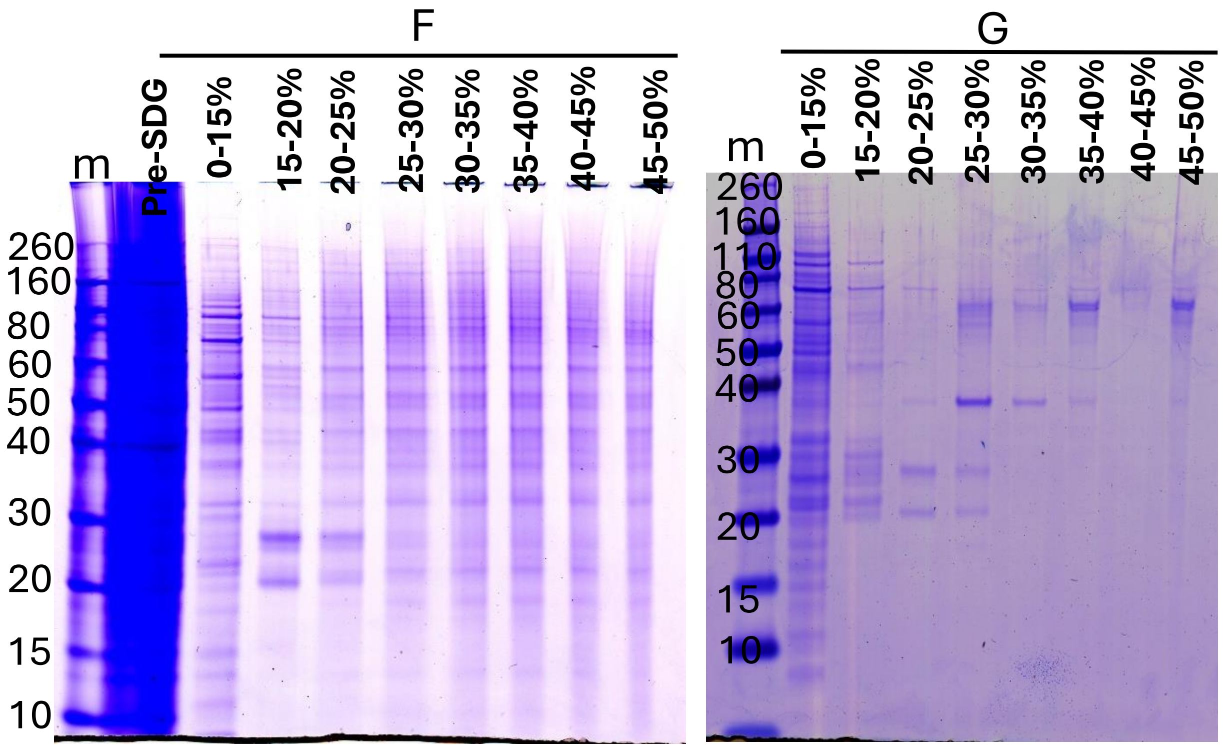

Figure 8B – western blot

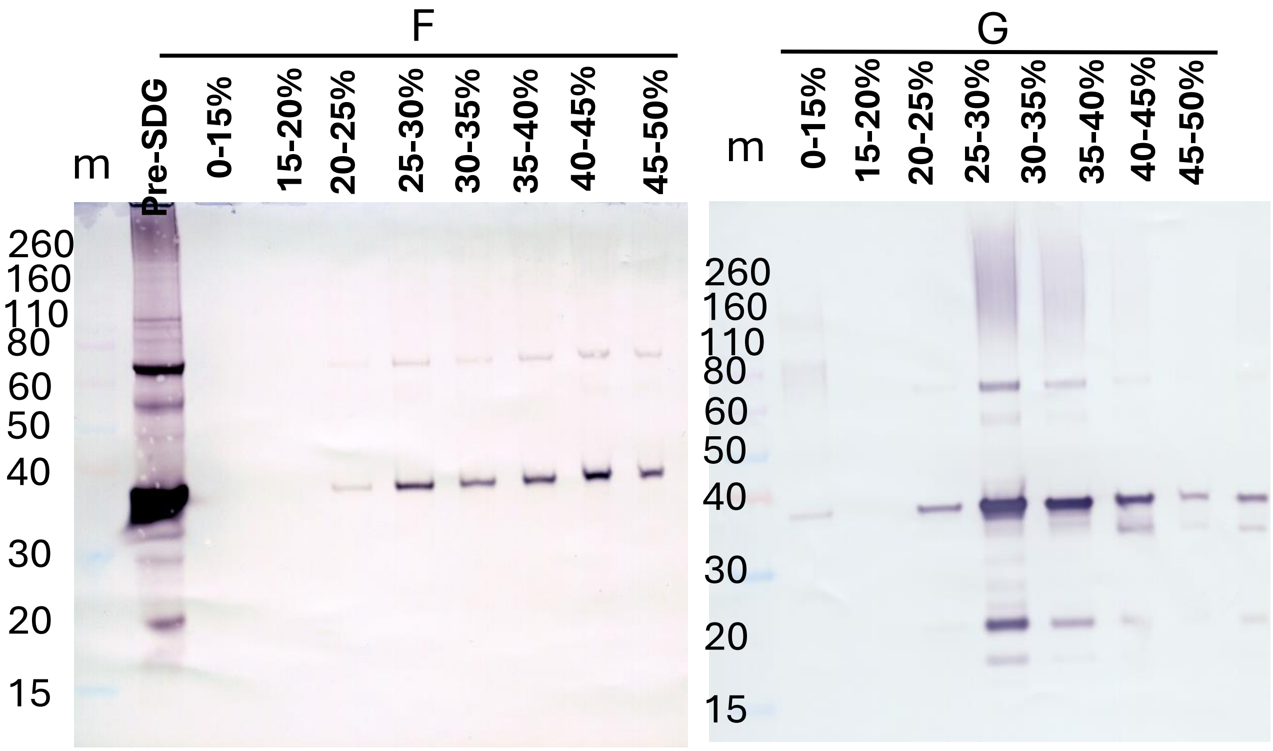

Supplement: Supplementary file 1 [file pathogens-14-00118-s001.zip › pathogens-3398703-supplementary.pdf]
